# How to conserve dignity in palliative care: suggestions from older patients, significant others, and healthcare professionals in Swedish municipal care

**DOI:** 10.1186/s12904-019-0393-x

**Published:** 2019-01-24

**Authors:** Ulrika Östlund, Karin Blomberg, Annika Söderman, Carina Werkander Harstäde

**Affiliations:** 1 Centre for Research & Development, Uppsala University/Region Gävleborg, 80188 Gävle, SE Sweden; 2 Department of Public Health and Caring Sciences, Uppsala University, Uppsala, Sweden; 3 Faculty of Medicine and Health, School of Health Sciences, Örebro University, Örebro, Sweden; 4 Centre for Collaborative Palliative Care, Department of Health and Caring Sciences, Linnaeus University, Kalmar/Växjö, Sweden

**Keywords:** Care actions, Community nursing, DCI-SWE, Dignity, Dignity care intervention, End of life care, Palliative care, Person centred, Sweden

## Abstract

**Background:**

An essential aspect of palliative care nursing is to conserve the dignity of the patient. A Dignity Care Intervention (DCI) has been developed in Scotland to facilitate this role for nurses. The DCI is now being adapted to a Swedish context (DCI-SWE) and a central step is to identify culturally relevant, dignity-conserving care actions. These care actions will be incorporated into the DCI-SWE. Therefore, the aim of this study was to suggest care actions for conserving dignity in palliative care from the perspectives of the patients, significant others (SOs), and health care professionals (HPs) in municipality care in Sweden.

**Methods:**

This study used a descriptive design with a qualitative approach. Data from 20 participants were collected through semi-structured individual interviews with patients (*n* = 3), SOs (*n* = 4), two focus groups with nurses (*n* = 9) and one focus group with physicians (*n* = 4) in two Swedish municipalities. These data were deductively analysed using qualitative content analysis with the Chochinov model of dignity as framework.

**Results:**

With the Chochinov model of dignity as a framework, care actions based on suggestions from the participants were identified and presented under three themes: Illness related concerns, Dignity conserving repertoire, and Social dignity inventory. The study found both specific concrete care actions and more general approaches. Such general approaches were found to be relevant for several dignity related issues as all-embracing attitudes and behaviours. However, these general approaches could also be relevant as specific care actions to conserve dignity in relation to certain issues. Care actions were also found to be linked to each other, showing the importance of a holistic perspective in conserving dignity.

**Conclusions:**

As part of the adaption of the DCI from a Scottish to a Swedish context, this study added relevant care actions for collaborative planning of individualised care in mutual dialogues between nurses and those they care for. The adapted intervention, DCI-SWE, has the potential to help the nurses in providing palliative care of evidence-based quality.

## Background

Dignity is a human right [1] and one of the highest priorities in healthcare [2–4]. The support of patients with palliative care needs to live with and die with dignity is an essential part of nursing care [5]. It can be argued that dignity is especially important in palliative care because of the vulnerability and dependence of the persons cared for [6]. The Swedish concept *värdighet* (dignity) has been described by Edlund et al. [7] as an entity consisting of body, soul, and spirit. Absolute dignity refers to values that are impossible to forsake e.g., human worth, freedom, responsibility, and serving one’s fellow humans. In contrast, relative dignity is influenced by culture, and has hierarchical and flexible values. Barclay [6] suggests that dignity in the contemporary era is associated with equality in worth. For a person within the healthcare setting who is severely ill and frail, dignity is about his/her ability to live in accordance with standards and values [6, 8] fundamental to his/her equal status as a person. Consequently, treating a person with dignity within the healthcare system involves supporting his or her capacity to uphold standards and values, and thereby avoid humiliation and shame [6].

Nurses can confirm the individuality of patients by reflecting upon what the unique person reveals. This implies responsiveness to and respect for the problems, needs, and desires expressed [9–11]. Dignity in caregiving requires individualised care, restoration of control, respect, advocacy, and sensitive listening [12]. Other factors that promote dignity are a culture of care, attitudes and behaviour of staff, and the performance of specific care activities [13]. Perceptiveness, openness, listening and respect are part of a comprehensive approach to dignity-conserving care for patients, alongside more concrete and specific care actions [10].

**Fig. 1:**
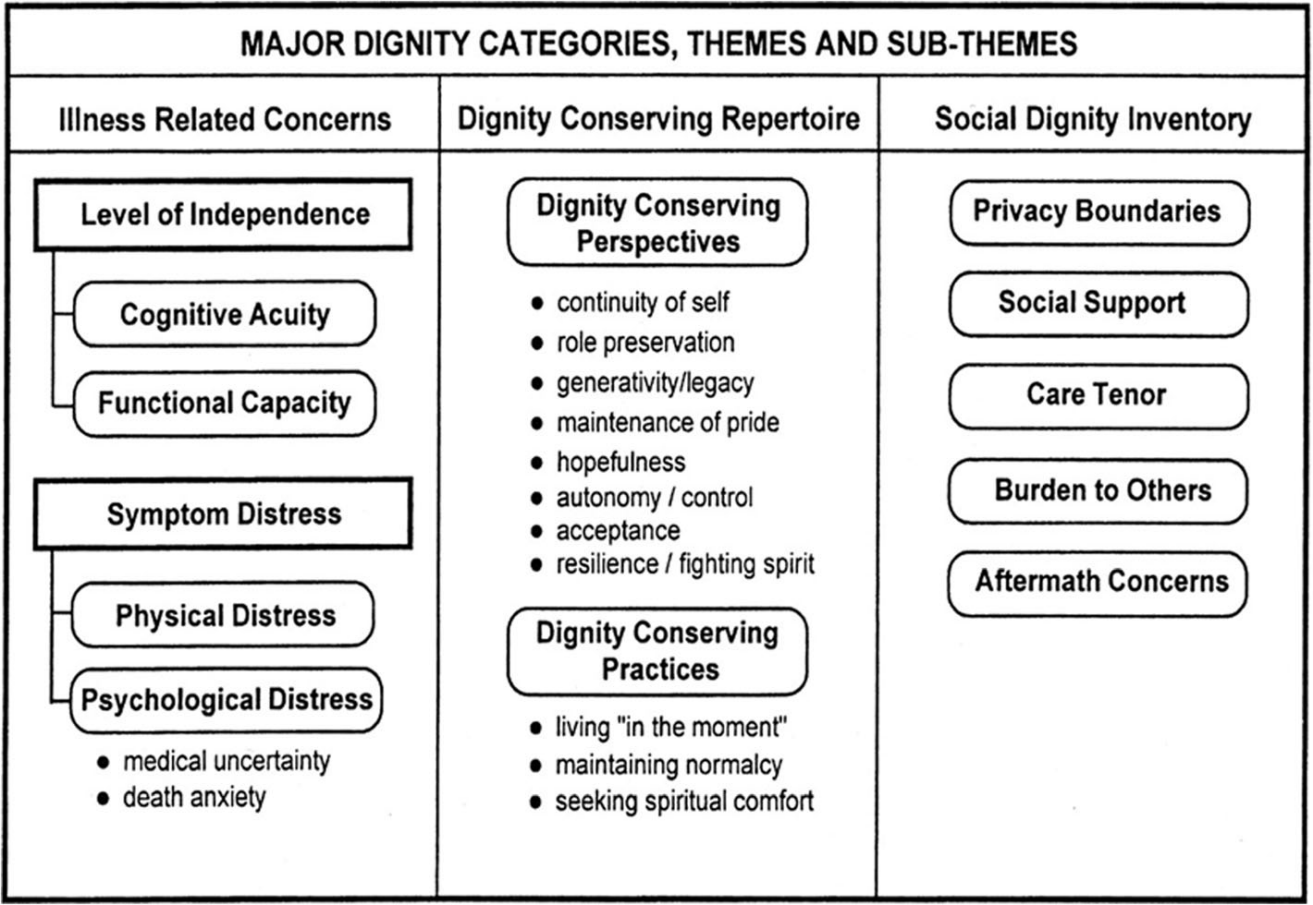
The Chochinov Model of Dignity [19, 20] with permission from the developer

Nurses have an obligation to work in a way that conserves the dignity of the persons they care for, but it is not explicit what this requires [13]. The nurses’ responsibilities and professional knowledge have to be facilitated in this regard [12, 13], but the National Guidelines in Palliative Care in Sweden [14] do not give much guidance. However, a Dignity Care Intervention (DCI) has been developed in Scotland by Johnston, Östlund and Brown for use by nurses caring for persons with palliative care needs [15–17]. It is an intervention that can give nurses guidelines for conserving dignity [6, 18]. The DCI is theoretically based on Chochinov’s model of dignity [19, 20] with its three main categories – illness related concerns, the dignity conserving repertoire, and the social dignity inventory. These three categories have respective themes and sub-themes, see Fig. 1. The model has been validated [21] and is empirically supported by factor analysis [22]. As such, the DCI covers a range of dignity-related concerns, including physical, psychological, social, and existential issues. Community nurses in Scotland and Ireland have found that the DCI helps them identify the dignity-related needs of their patients and provides a holistic, person-centred care at the end-of-life [16, 23]. Patients have also described that the DCI enables them to engage in dialogues with their community nurses about important issues that they might not otherwise raise [24].

The DCI consists of several components [24]. The DCI provides an education programme and instructional manual for its implementation. A screening tool, the Patient Dignity Inventory (PDI) [25, 26], is used to identify patients’ dignity-related concerns. Thereafter, reflective questions are used in a dialogue between the nurse and the patient to deepen the understanding of the patient’s concerns and preferences for how to deal with them. Finally, suggestions of specific care actions– to conserve the patient’s dignity in relation to identified concerns – are used in accordance with the patient’s preferences. The circle of care is continued by the reuse of the PDI and reflective questions to evaluate the response to care actions and to identify potential new issues requiring care actions [24].

Care actions for the DCI were derived from reviews of international research literature [17, 27], and from focus groups with patients, significant others (SOs), nurses and physicians in Scotland [15]. Multiple types of input were used because effective, evidence-based nursing should be drawn from a range of sources [28]. The use of DCI has been studied in Scotland and Ireland [16, 23, 24] but not yet in Sweden as no Swedish version exists. We are currently adapting the DCI to fit a Swedish context, following a similar method approach as where it was developed, with the addition of language translation. A review of Swedish research literature has been conducted [10] and will – together with this study – provide culturally relevant care actions for a Swedish DCI (DCI-SWE). The aim of this study was to suggest care actions for conserving dignity in palliative care from the perspectives of the patients, SOs, nurses and physicians (health care professionals, HPs) in municipality care. These will be part of the DCI-SWE.

## Methods

A descriptive design with a qualitative approach [29] was used. Data were based on individual interviews with older patients and SOs as well as focus groups with HPs. Data were deductively analysed using qualitative content analysis [30] with the Chochinov model of dignity [19, 20] as framework. The model’s themes and sub-themes [19, 20] are hereafter referred to as themes. See Fig. 1.

### Setting and sampling

The study was conducted in two municipalities in Sweden that included both rural and urban areas. A purposeful sampling [31] was used to recruit patients, SOs and HPs, until sufficient coverage of aspects of the model was achieved during the interviews and in the focus groups. Inclusion criteria for patients were that their care providers considered them to have palliative care needs. Inclusion criteria for SOs were that they were close to such a patient. Exclusion criteria were an inability to understand Swedish and/or cognitive impairments. HPs could be included if they were working regularly with palliative care. Patients and SOs were identified by the municipal palliative care coordinators, and then contacted by a researcher to arrange an interview. HPs were identified either through a Centre for Collaborative Palliative Care, by a medically responsible nurse in a municipality, or by a municipality palliative care coordinator.

### Data collection

The interviews and focus groups were semi-structured [31] and followed the interview guide used for the development of the original DCI [15], translated to Swedish. The questions covered all themes in the model of dignity [19, 20]. Interviewees were considered experts and asked to provide examples, e.g., ‘In your view, how can persons receiving palliative care best be supported to manage feelings of being a burden to others?’ Questions such as ‘Can you explain this further’ were used to probe their answers. Patients and SOs participated in individual interviews to capture their individual thoughts and suggestions [32]. These interviews were conducted by one researcher (AS or UÖ with long experience of meeting and communicating with patients in palliative care), at a place chosen by the participants. They had no previous relationship with the participants. HPs participated in focus groups to facilitate not only individual suggestions, but also to obtain clarification of their views through group interaction [33]. These focus groups were led by one moderator (UÖ) and a co-moderator (CWH or KB), all with training and experience of performing focus groups. Most of the participating HPs knew about the DCI-SWE project and knew at least one of the researchers before the focus groups. All interviews/focus groups lasted 60 to 90 min and were audio-recorded.

### Data analysis

All audio-recordings were transcribed verbatim by a professional transcriber and checked against the recordings by the interviewers. The transcribed data were deductively analysed using qualitative content analysis [30]. The themes in the Chochinov model of dignity [19, 20] were used as a framework for structuring the suggested care actions to conserve dignity. The first and last authors (UÖ, CWH) searched the text for care actions that fit the predefined framework. These text units were marked, then condensed and assembled into a new document. They were thereafter labelled with a code based on their content. The coded units were then sorted according to the themes in the model and described in text. If there was difficulty in classifying a care action, the first and last authors discussed it until a consensus was reached. The second and third authors (KB, AS) were involved if divergent opinions occurred. Throughout the analysis, discussions were held about what actions qualified as “concrete care actions” for dignity related concerns. Finally, the synthesised suggestions on care actions were checked by the second and third authors for adequacy of the care actions and their placement under the different themes. In the result section, the source of the suggestions i.e., patients, SOs, HPs, is indicated in parentheses. To ensure trustworthiness, participant quotations are shown in Tables 1, 2 and 3.

**Table 1:** Quotations about the theme “Illness related concerns”

Cognitive acuity	*“There is a need to create a calm, harmonious environment with plenty of time and few distractions” (Physician)*
	*“A clarity in the information given so that they can follow the line of thought and understand” (Physician)*
	*“We have to pick up, we lead them, remind them. It is our duty as nurses to give a bit of structure” (Nurse)*
	*“You simply have to say that it is part of the illness, of the whole illness situation, because you are shocked by everything and you are shocked by how people view you” (Patient)*
Functional capacity	*“You might have to reconsider. Maybe today the goal is to take a shower and try to do that on your own. Maybe you need to rest before that and not plan anything else” (Nurse)*
	*“That you ask the person how you want to be supported. Do you want healthcare professionals to come and help you, or do you want to try and do it as well as you can on your own?” (Patient)*
Physical distress	“*To affirm the distress, that you see the symptoms, and that you see that it is hard. To lift it to the surface and not hide it” (Nurse)*
	*“To be safe, and to have the assurance of knowing whom to turn to and not having to wait for hours on the telephone to talk to the healthcare worker. That does not work when you are ill; you want an answer now and not in five hours” (SO)*
Psychological distress	*“To affirm the feeling that we do not have all the answers. Sometimes shit is shit I used to say. Sometimes it is miserable. Then you must be allowed to say: ‘Yes this is the way it is, so I understand that this is horrible for you.’ You can affirm the feeling, not just finding solutions. Because sometimes, I believe, it can be stressful if we say: ‘No, it is not so bad, we can do this and we can do that.’ Sometimes shit is shit.” (Physician)*
	*“You have to take this very seriously... because you do not say everything directly to the healthcare professionals” (Patient)*
Medical uncertainty	*“We rumble in, we go through the medicine lists, we examine and we talk. But how are they doing when we have left? That is when they need continuity and recurrent regular information” (Nurse)*
	*“They need conversations, as a help to put words to what concerns them” (Nurse)*
	*“I think it is important that you ask the patient, what do you want to know, what do you reflect on? Sometimes we think we know what the person does not know, but it might be something totally different that they want to talk about” (SO)*
Death anxiety	*“A lot of information is needed. To have a dialogue concerning what is happening, to make it a process. There is a lot of safety in that they know and that they are prepared, that they know a lot. I believe that understanding and information are very important. Imagination is often worse than reality” (Physician)*
	*“The most common question is: ‘Will I be in pain?’ Then it can be comforting to talk about that there are medications for this” (Nurse)*

**Table 2:** Quotations about the theme “Dignity conserving repertoire”

Continuity of self	*“Learn to know the person. To talk about, and have knowledge, of their life and history. That one relates to what they have been and sees them as that person. Show interest and curiousity” (Physician)*
	*“That one does it in consultation with the ill person. That one initiates a conversation about how they want to plan their day…” (Nurse)*
	*“Well, the whole time since he became ill, I have told him, ‘You have to tell everyone how it is, it does not need to be in detail, but tell them how it is so you can get much more support’. Otherwise people speculate, why do you look like this, why it is so…. If one can, and has the strength, I think one should be open” (SO)*
	*“Simple support, that gives security even if not used when one does not need it… I have been a little doubtful regarding home care because one wants to be yourself at home, one want to be the same as before” (Patient)*
Role preservation	*“That they participate in daily issues like paying the bills or calling the plumber if the furnace breaks down. Maybe also include relatives – it is very important not to exclude the one that is ill” (Nurse)*
	*“People that I know – they are not in palliative care but they are seriously ill – they have their interests. One of them likes to bake and others have other interests. One should, sort of, put them in that direction” (Patient)*
	*“At home we have tried him cooking the daytime meal, that is his task, and he has done some smaller renovations around the house....Then he feels that he has something to do, to unburden… he has taken responsibilities for some things that are not usually his tasks, so we have had to shift some roles, actually” (SO)*
	*“Well, when there are children, that one does not have to cope with shouldering the parental responsibilities. Maybe the children do not want to visit their parents as they find it hard. Then one can talk about it. What is possible, what is reasonable, how can you as a parent try to help, for example, a teenager. Perhaps they can visit an hour now, to keep up some kind of a relation, and then go outside, to play and jump. One can be supportive in such things, just by helping them frame their situation” (Physician)*
Maintenance of pride	*“Some reassurance, as they can be very glad to realise they can still manage things although they might not think so at the time” (Nurse)*
	*“So that one sees the patient the whole way through, and this is what the patient needs help with” (SO)*
	*“That the physician says, ‘I understand you are in pain’, instead of saying, ‘You are very alert for your age’, when I do not feel like that. I have pain here and there and distress, dizziness… ‘But there is not really anything wrong with you, you just make things up’… Well, yes, being acknowledged and listened to, it does not usually help saying, ‘Oh you are so alert today!’ I am actually in pain, I am over 90 years old and having pain and no one seems to care about me having that” (Patient)*
Hopefulness	*“As long as you can, you do things. For example when I go out cycling, I am so glad that if I can cycle down to Willys and shop. I think it is not too far and even if I will get tired, I get some company there... Then when I get home I can think, this was actually nice. But I have realised the situation. I do not go out shopping new clothes. I use what I have, and then…[it’s] like keeping death away” (Patient)*
	*“One has to look back on what one has accomplished and dream back. Enjoy a long life lived that has been rich. Then that it is coming to its end and one does not have the strength to do things as before… it is good to be able to look at photos, on the phone, computer or Ipad” (SO)*
	*“Focusing on the small goals and meanings in life. That one has a bearable situation in the time still left. It is about* Carpe Diem*. It might be meaningful and a goal to get out in the autumn sun for a while during this afternoon. It is the smaller world that becomes the goal and meaning. That I manage to meet my grandchildren for a short while. A kind of hope having a small goal. A small and feasible goal” (Nurse)*
	*“And then you talk about the time to a wedding, or to midsummer eve. The man who participated in the midsummer festival in his town for many, many years, he asked me directly: ‘Will I make it to midsummer eve?’ I said yes and then he engaged in his usual chores. Well that is a way to find hope, not thinking too far ahead…” (Physician)*
Autonomy/control	*“It is difficult, it is tough, to see as a relative or staff, and one has to be very humble to the person och try to ask as much as possible about how they want to have things… so that one can, somewhat, keep them involved even if they really can’t and that one has to take help from relatives to make it as good as possible” (SO)*
	*“Even if the doctors want the best for you, it is not always possible, so I brought that up with the physician. Now it is my quality of life not theirs, irrespective of how well they mean” (Patient).*
	*“That is a question one asks the patient, ‘what do you need to feel you are in control? What is important for you, and perhaps one cannot control everything’… But there might be small parts, such as physical things like coping with pain or wetting oneself, not wanting your wife to see you are in pain, or such things. But they are different things and they overlap. What is important for the person is key. But all parts that we have talked about are needed – the dialogues, symptom relief, aids, everything” (Physician)*
	*“One tries to let them keep control over their lives, the staff cannot take over everything, I think that happens: ‘now you will receive lots of help from us’, and all of the sudden one feels that it is the staff that decides over everything. Sensitive listening… that it continues to be as before, when I want to have my evening tea, when I want to go to bed, the TV program I want to see... one has to adapt to that” (Nurse)*
Generativity/legacy	*“In their homes, when one sees things, perhaps an interest they have had, something they may have built, some handicraft – one sees this is a person that likes to embroider. Then one can talk about it and ask about that. I mean they feel seen as persons, not as a patient being in pain again” (Physician)*
	*“Sometimes it is like, one is too much here and now. One forgets that there has been a history that is also important. It is important that the staff talk with patients and relatives, what have they done before and how their lives have been. Not just seeing the person in front of you who is ill, but a person that once was well, as well as I am” (SO)*
	*“We once had a patient that had some unfinished business and when she got closer to the end, she felt terrible. She told this to someone, it was something about a son she really wanted to talk with, and then we helped, because she didn’t have any relatives living close by. We helped to get in contact with this son and he came and they met and then, it was just like she got peace., She had such anxiety before that it was almost impossible to relieve” (Nurse)*
	*“When you have let someone down, [it is good] having someone that can help you to unravel it a little. Well we can always do this, instead of saying ‘do not think about that’. It is just that this is what is usually done” (Patient)*
Acceptance	*“It is the quality of life, and as a palliative patient one has to take that in, that it changes. It doesn’t go away, one is not just as good at things anymore” (Patient)*
	*“People are very frightened, even those dying. It is very important, in that sense, to think it through” (Patient)*
	*“Well it is easier when the communication is good, when it is somehow straight and honest from the beginning, that makes it so easy. That is something one has seen – that it is better telling” (Nurse)*
Resilience/fighting spirit	*“That they have accepted they are dying, and can say, ‘I know I am dying’. Then one supports them during this time until they are to die. To work for them feeling that there is still some meaning and quality of life… Well there are various patients and individuals, so one cannot generalise and do the same with everyone, but always take into account the individual… Lift them up and sometimes dare to laugh and make jokes” (Nurse)*
	*“…first of all I think one must be genuinely honest and tell how it is even if it feels heavy, so that one knows. Then it is a question, when it comes to medication, what is it that one gets and the effects. If one will receive painkillers, what will it mean, is it so that one will be even more tired or drowsy? One has to be very clear, inform when one does things, what it means, and the effects” (SO)*
	*“Well, one has to cycle with fewer groceries and such things, as I have a problem with my balance, but I can still experience dignity by shopping myself” (Patient)*
Living in the moment	*“That one should help them to replace and create new routines, give suggestions that maybe you can do like this instead, what do you think, could this work? This might also be a way to influence the situation. Okay, this is how it is and it may be just as good if I do it in another way” (Nurse)*
	*“So one needs some long term planning, but perhaps not planning too much as we don’t know if it can be done next week as then he might not have the strength or something else happens… Well, however one tries in life, everything can be totally messed up” (SO)*
	*“Well, one has to be glad that there are people that can help. That is how I feel about the home care, it feels safe, and I got it explained to me: ‘We will come, we will be there for you’. That felt so good” (Patient)*
Maintaining normalcy	*“From the moment we got there, we were very well looked after, both us the relatives and mum. Also dad could come and stay for mum’s last days. They took her up in a wheelchair, so she could participate in having breakfast even if she did not eat much, but she could still be up” (SO)*
	*“To be able to have some fun today, one might need help with ADL to keep the strength for doing the fun stuff” (Nurse).*
	*“My daily routines have been quite simple…[do you have support from your wife in accomplishing them?] Yes, it is thanks to her I am still alive…” (Patient)*
Finding spiritual comfort	*“To help them get in contact with for example, a deacon. First and foremost raise the question, and ask if they need help, volunteer help. We have a list of priests that we can contact…” (Physician)*
	*“When a patient is assigned to a care team one must ask, in some sort of way, ‘Do you have a faith, do you belong to a church or how do you think of a life after this?’ That gives you a hint. ‘Are you member of another church or an atheist, have you left the church or do you have another creed?’ One should not be afraid asking. Just as you ask about pain, you can also ask about faith…” (SO)*
	*“Yes this came up when the contact nurse was here. And if you are at a hospice, there is a hospital church. You can just ask to get to talk with the priest, it cannot be too difficult. And if you belong to another church of some kind, why not ask the nurse, ‘Can you call the pastor or the deacon?’ Deacons are good on talking, they say” (Patient)*
	*“[Your mentioned before that one can get consolation from nature] Yes, definitely… One hopes that there are some relatives or friends that can take you out in a car and maybe stay at a rest area. Then one does not do much more than sit there if they are having difficulties walking or such things. Because, nature has always been important for me, especially if one’s thoughts are dark….” (Patient)*

**Table 3:** Quotations about the theme “Social dignity inventory”

Social support	*“They should not feel that they are left out, absolutely not. They should not be hanging in the air. There should be someone that they can turn to…a phone number or something, so both the relatives and the patient know [who to turn to]” (Nurse)*
	*“Those who have relatives and friends, they are lucky. They have someone who can speak for them, when they cannot do it themselves” (SO)*
	*“You need to find those who need this extra support so you do not give the same support to everyone” (SO)*
Private boundaries	*“If you are at a hospital or residential home, then the room or apartment is the patient’s. You are the guest. You knock on the door before you enter” (Nurse)*
	*“And if a healthcare professional happens to be here when I get a phone call … then I might say, ‘Can you call me later?’ That is a way to protect your private life” (Patient)*
	*“It can be difficult at a hospital because it is the healthcare professionals’ territory. They stomp into the room* e.g.*, to fill up the clean laundry. They do not think that this is a private space” (SO)*
Care tenor	*“I met a patient today who told me that the home care service was in too much of a hurry. He was in pain and they did not listen. Then you need to have a meeting and discuss this. That is the way you have to try to handle it. If you can do something about it, you must try” (Nurse)*
	*“It is important to have respect, that you talk to the person involved and not just go in different directions” (SO)*
Burden to others	*“To make them talk to each other [patient and relative]. To face the feelings and tell each other how they feel. Because it might not be that way [that the patient is a burden] and then the relative can convince the patient that that is not the case, it is just the patient who feels that way” (Nurse)*
Aftermath concerns	*“And sometimes the patient has a wife who has dementia or he knows that she will not be able to take care of herself. Who will take care of her? Then you need to step in and inform that we will do that, there is help and we will take care of it so that the patient can be confident in that” (Nurse)*
	*“What worries me is how my wife will have it afterwards, if we should have sold the house earlier and gotten an apartment instead. But she thinks it is hard to imagine herself living in an apartment” (Patient)*

## Results

The findings were based on data from 20 participants (three patients, four SOs, nine nurses and four physicians). The patients were two men and one woman, 71 to 80 years old, of which two had cancer diagnoses and one had severe heart disease. All were receiving palliative care, either at home or in a nursing facility. The SOs were all female, between 53 and 86 years old. They had a close relationship to a patient in palliative care but not to any of the patients included in this study. Three focus groups with nurses and physicians were conducted. These were not involved with the three patients in the study. One group consisted of six registered nurses, all of whom were working within the context of home care in a small Swedish municipality. A second group included one registered nurse with a special responsibility for the palliative care in a home care unit and two registered nurses with overall responsibility for coordinating palliative care in a large municipality. The nurses were all females with 6 to 35 years’ nursing experience. They were specialised as: district nurses (*n* = 3), in palliative care (*n* = 1), critical care (*n* = 1), oncology care (*n* = 1), geriatrics (*n* = 1), leadership (*n* = 1), or without a specialist training (*n* = 1). The third group consisted of physicians, two females and two males, with long working experience (15 to 35 years). All had been working, from 1 to 13 years in specialised palliative care, either as part of a palliative consulting team or in oncology care.

The findings are presented under the themes in the framework. A short explanation of the theme is given [19, 20], followed by the results of suggested care actions.

### Illness related concerns

*Illness related concerns* are difficulties related to illness that can threaten or influence patients’ experience of dignity. They involve two themes: *Level of independence* and *Symptom distress* [19].

#### Level of Independence

*Level of independence* concerns to what degree help is needed from others and is characterised by the ability to maintain one’s mental capacity. It covers *Cognitive acuity* and *Functional capacity* [19].

In regard to *Cognitive acuity,* patients’ ability to keep their mental capacity and thinking could be facilitated by supporting their own resources, e.g., by creating a plan in collaboration with them. It is important to approach the patient when he/she is most alert and to use simple, explicit everyday language to facilitate understanding (HPs). An open dialogue to learn why patients were sometimes not clearly understood was also suggested (patients). Involving SOs improves understanding of the patient’s wishes and healthcare professionals should support SOs to manage the course of events (SO, HPs). Rating scales using images of symptoms, instead of just text, could influence cognition as well as medical treatment (HPs). It is important that nurses and physicians assess cognitive acuity instead of just assuming patients are unable to think clearly (SOs).

In regard to *Functional capacity*, nurses and physicians should be cautious when approaching the patient bodily and intellectually (SOs). A need for sensitivity towards patients who had difficulty accepting that they could not manage their daily living was emphasised (SOs, HPs). Patients should be encouraged in recognising accomplishments and surmounting of challenges (HPs). Healthcare professionals should support the patient in daily living (HPs), and plan forward in joint agreement with the patient and maybe also with the SOs (patients, HPs). Accepting help is sometimes easier for the patient if the help comes from SOs (patients). To balance patients’ needs and demands with abilities and energies of the SOs, the SOs sometimes needed to be prioritised over the patients. For patients living in their own homes, securing availability of care was suggested (HPs).

#### Symptom distress

*Symptom distress* is characterised by *Physical* and *Psychological distress* with the latter comprising *Medical uncertainty* and *Death anxiety* [19]. This emphasises the need for healthcare professionals to be well acquainted with the patient’s anamnesis (SOs).

Concerning *Physical distress,* confirmation of patients’ feelings and giving hope of relief were suggested. It was also suggested to try one thing at a time to relieve distress and evaluate its effect in a calm and safe atmosphere (HPs). SOs were suggested to be sensitive to patients’ symptoms and act as patient advocates when the patients are too weak to act on their own behalf (SOs, HPs). Healthcare professionals with competence and authority were requested, as was continuity in contact with the care facilities (patients, SOs).

Concerning *Psychological distress*, healthcare professionals are to be present, listen, talk, give time, confirm the patient’s feelings, and reassure them that they are not alone in their situation (HPs). What is said and what is not said should be noticed (patients, HPs). SOs need to be supported in a similar way (SOs). Mood enhancing drugs were suggested for treatment of overwhelming feelings (HPs), as were different ways to distract feelings of despair e.g., taking walks (patients).

To ease *Medical uncertainty* for the patient***,*** healthcare professionals should inform them about their availability and create possibilities for perceptive dialogues about their worries (HPs). The healthcare professionals should explain about the disease and examinations, and what is happening step by step (patients, SOs). If desired, this could take place with the SOs present (SOs). Healthcare professionals need, however, to be aware that patients might not wish to talk about their forthcoming death, or they might choose to talk to someone else about it (HPs). Conversations should be held privately with sensitivity towards what the patient and SOs want to talk about (patients, SOs).

Good relations with both patients and SOs are essential when talking about *Death anxiety.* Perceptiveness is important as the need to talk can be explicit or proceed from implicit worries and vague cues (HPs). Some patients and SOs wish to participate in conversations about death and dying, but others do not want to share their thoughts in this regard (patients, SOs). A calm safe environment can contribute to making these conversations easier. Assurances that medications are available to relieve pain can prevent worry and anxiety (HPs).

### Dignity conserving repertoire

The *Dignity conserving repertoire* includes those aspects of patient psychological and spiritual landscape which influence their sense of dignity. This repertoire is divided into the themes *Dignity conserving perspectives* and *Dignity conserving practices* [19].

#### Dignity conserving perspectives

*Dignity conserving perspectives* refer to those ways of looking at, or coping with, one’s situation that influence the sense of dignity. It is described by eight sub-themes [19].

*Continuity of self* refers to a sense that the essence of whom one is remains intact [19]. Support of the patients in adapting to their new functional levels was suggested (patients). Facilitation of the patient’s activities should be done in collaboration with the patient (HPs). Through listening, healthcare professionals can learn to know and acknowledge the person behind the illness, notice the patients’ interests, and initiate conversations around them (HPs). For patients who feel their appearance has changed significantly due to their illness, their desire for privacy in this regard should be respected. This could mean not parking an identifiable car immediately outside the patient’s residence, respecting the patient’s wish not to use aids in the presence of others, helping them to obtain a wig or breast prosthesis, or offering institutional care for those that want to “hide” (HPs). However, encouraging patients to be open about their situation was also suggested (SOs, HPs). Patients may hesitate to accept help at home to retain their prior sense of identity and to continue feeling useful, but the availability of home care can add a feeling of security (patients).

*Role preservation* is about the ability to remain invested in one’s usual roles, as a way of maintaining congruence with a prior view of oneself [19]. It was suggested that patients should be enabled to do things they want and still have the strength to do. This could be done, e.g., by adequate medication, involvement of SOs, and availability of home care. Patients should be supported in adapting or terminating roles (patients, SOs, HPs). Patients and SOs with young children should be supported in their parental roles (HPs). Care actions to support patients in the continuation of their social lives were suggested e.g., by encouraging them to let those around them know about their illness, facilitate them with aids, and support their SOs in getting the patient to social events (patients, SOs, HPs).

*Maintenance of pride* is about the ability to maintain a positive sense of self-regard or self-respect [19]. The importance of being able to do valuable things and of being a source of joy to others was stressed (patients). This can be enabled by supporting daily activities (ADL) to save energy (HPs), while at the same time respecting patients’ wishes for independence (patients). Healthcare professionals should be empathic and see patients as persons with capacities, who only want help with things they really need help with. SOs and healthcare professionals should take the patients seriously by acknowledging them instead of ignoring them (patients).

*Hopefulness* is seeing life as enduring, as having sustained meaning or purpose [19]. If the patient cannot find meaningful things to do, it was suggested to look back on what they previously enjoyed in life, e.g., through narratives or photos. Furthermore, one should focus on “small” goals that could be accomplished and support the patients in achieving them (patients, SOs, HPs). SOs should be kept involved, as they can be a source of meaning for the patient. Healthcare professionals can talk with SOs and patients about joyful things they may be able to do together, but also acknowledge the normality of being sad together. Furthermore, healthcare professionals should be involved in dialogues with suffering patients and, for those patients who want to talk about the time left, be honest about it being limited and supportive in planning for it (HPs).

*Autonomy/control* is about the ability to maintain a sense of control over one’s life circumstances [19]. Patients can take control by doing achievable things (patients) and SOs may be involved in helping them accomplish activities (SOs, HPs). Dialogues were suggested about what is important for the patients’ sense of control. It was also suggested to support the patients in being realistic about their hopes that things should be as before (HPs). It is important to involve patients in decisions and support them to share how they want to be cared for and where they want to die (SOs, HPs). Healthcare professionals are suggested to advocate for patients when they themselves cannot manage, and this includes being honest towards the SOs (patients, HPs). Other suggestions included symptom management, care aids, continuity, and respecting the patient’s home as a home and not primarily a care facility (HPs).

*Generativity/legacy* refers to the solace or comfort of knowing that one will leave behind something lasting that transcends death [19]. Supporting patients by talking with them about their life (HPs) and precious memories was suggested. So were facilitating them to have photos and memories around, writing diaries, and staying in contact with friends and family (patients, SOs, HPs). For patients experiencing that they have unfinished business, it was suggested to help them make a plan to accomplish closure (patients, SOs, HPs). Healthcare professional can facilitate contact with persons involved in these issues to enable them to unravel things in collaboration. When things cannot be sorted out, the team can help to balance this difficulty with remembrances of the positive experiences in the patient’s life (HPs).

*Acceptance* is about the ability to accommodate to changing life circumstances [19]. The necessity to accept that some things cannot be accomplished anymore, that they might need to be done in alternative ways, or with support from others, is emphasised (patients). Honest communication is suggested (patients, HPs) when patients are assessed to be ready for such conversations (HPs). For some patients it might be sufficient to talk to their SOs. It is also important for patients to know that healthcare professionals are available for them until the end (patients). True presence with the patients in their sorrow is suggested (HPs), as is the use of humour (patients).

*Resilience/fighting spirit* refers to the mental determination exercised to overcome illness or to optimise quality of life [19]. Dialogues are suggested that include sensitive listening to patients’ views on how they confront “the death” (patients, HPs) and the use of humour when appropriate (HPs). Information about illness and treatment should be given in a strict, honest way, tailored to make it understandable (patients, SOs), and preferably with the SO present (SOs). Patients that ask for a second opinion are to be helped with this by the healthcare professionals (HPs). Patients should strive to adapt activities to their functional level to support dignity (patients). Healthcare professionals are suggested to keep in regular contact with patients to facilitate problem solving (SOs).

#### Dignity conserving practices

*Dignity conserving practices* comprise a variety of approaches or techniques used to bolster or maintain a sense of dignity. These are presented as three sub-themes [19].

To *Live in the moment* means focusing on immediate issues to avoid worrying about the future [19]. Facilitating for patient to carry out everyday activities is suggested as this is linked to the patients’ ability to focus on the moment (SOs, HPs). It is important to seize the moment and patients can be supported with necessary aids (HPs). Keeping some long-term plans to look forward to, but at the same time being realistic that the plans may never happen, is important (SOs). The availability of support gives relief from worries about the future (patients).

*Maintaining normalcy* refers to the sense of continuous behaviour of performing usual routines and schedules while coping with the challenges of being ill [19]. It is important for patients to be in charge of some activities (patients). Healthcare professionals are suggested to support patients in everyday activities e.g., by planning the day with respect for the patient’s condition, by focusing on “small goals”, and by the performance of activities in alternative ways. Moreover, they need to be perceptive to patients’ habits (SOs, HPs). Helping patients with, e.g., getting dressed can conserve energy to be used for participating in things they enjoy on a daily basis (HPs). It is also suggested to involve SOs (patients, SOs). Care actions that enable the patients to stay in their home are also suggested (HPs).

*Finding spiritual comfort* is about the dignity-sustaining effects of finding solace in religious or spiritual beliefs [19]. Healthcare professionals are suggested to engage in conversation with the patient and explicitly ask about spiritual needs (SOs, HPs). Moreover, they can inform about available spiritual support and facilitate spiritual or religious practices that the patient is already involved in (patients, HPs). Spiritual comfort can also be found through culture, nature, and a positive outlook (patients, SOs).

### Social dignity inventory

*Social dignity inventory* refers to relationship dynamics and social issues that strengthen or weaken patients’ sense of dignity [19].

*Social support* is having access to a community of friends, SOs, and healthcare professionals who are present and helpful [19]. Dialogues are suggested to confirm the patients and SOs sense of involvement (patients, SOs, HPs). Supportive healthcare professionals explain unclear information, give advice, act as containers for feelings, and are present in death and dying. They coordinate contacts with healthcare (HPs) and sometimes need to act on behalf of patients and SOs who are unable to negotiate for themselves (SOs, HPs). Healthcare professionals could help create a network around patients and SOs to generate a feeling of control and continuity (HPs). Supporting the whole family can create security and strength for SOs to care for the patient (SOs, HPs). Room for visitors and possibilities for functional IT-technologies at care facilities can allow patients to maintain their relationships with family and friends (SOs).

*Private boundaries* concern how dignity is influenced by intrusions into personal circumstances in care situations [19]. There is a need to act respectfully and be sensitive to protect patients’ integrity (SOs, HPs). Thus, avoiding feelings of exposure during the patients’ routines should be respected and care should be tailored for each patient (HPs). Patients should have privacy and a private room if cared for at an institution (patients, SOs). When caring for patients at home or in residential homes, healthcare professionals should act as a guest e.g., knocking at the door before entering (HPs).

*Care tenor* refers to the attitudes that others demonstrate in interaction with patients [19]. Patients and SOs should be invited by healthcare professionals to talk about their concerns in a respectful atmosphere (HPs). It is important that healthcare professionals say hello and inquire how patients and SOs are feeling (SOs). The healthcare professionals should not be guided solely by routines but be respectful to the patient’s wishes and needs (patients, SOs). Collegial guidance on dignity, ethics and communication methodology is suggested for healthcare professionals so that they can successfully support patients and SOs and, when necessary, mediate in conflicts (patients, HPs).

*Burden to others* concerns the distress of being forced to rely on others for various aspects of personal care [19]. It is important to enable patients and SOs to be open with their feelings towards each other, to unravel misunderstandings, and to establish what resources are available within the family (HPs). Patients might be reluctant talking about their shortcomings and need for help, making it difficult to find solutions (SOs). The patients are suggested to lower their demands on what is to be done at home and instead engage in joyful activities together with the family. Patients also need to accept that not everyone in their extended family can manage to engage with them (patients). Patients’ and SOs’ wishes are to be recognized and healthcare professionals have to be perceptive to vague expressions. Help should not be imposed without learning what the family wants. Offering respite care – to ease the situation of the SOs and to relieve the patients from feeling as a burden – should be presented in a positive way to prevent the patients from experiencing themselves as even more burdensome (HPs).

*Aftermath concerns* refer to the concerns that the patients have about the effects of their death on those left behind [19]. Here SOs should be engaged so that patients know that things are sorted out before they die. Professional expertise might be necessary if younger children are involved. Sometimes there might be need to “give permission” to die to the patient who is resisting death due to unfinished business (HPs). Conversations are suggested about how life will go on after the patient has died, in joint consultation with the patient and SO (patients, SOs). Patients can also be encouraged to create memories of themselves for the family to keep (HPs).

## Discussion

In this study, we propose dignity-conserving care actions, from the perspectives of patients with palliative care needs, SOs, and HPs. This is part of translating and culturally adapting the DCI to a Swedish context. In the development of the original DCI, qualitative data were collected in Scotland [15] that together with reviews of international research literature [17, 27] formed the original care actions. For the adaptation into the DCI-SWE, we followed the same approach. The Swedish research literature was reviewed [10] and qualitative data from different stakeholders were collected which this study result is based on. The suggested care actions in the result included listening, communication, information, symptom control, facilitation of daily living, and participation in decision-making. Similar results were also found in the literature review [10] and thereby provide a solid ground for suggested care actions.

In the current study, the review of Swedish research [10], and the studies conducted for the development of the original DCI [15, 17, 27], we found both general and specific care actions. The embracing attitudes and behaviours to conserve patient dignity are related to what Gallagher et al. [13] report regarding dignity in the care of older persons, namely, the persons should be cared for as individuals. The results in our study also elaborate on what Hemati et al. [5] found to be attributes of dignity in palliative care, namely respect for privacy, spiritual peace, and hope. Here our study suggests explicit care actions to handle these concerns. Thus, the findings in our study coincide with Chochinov’s thoughts about dignity conserving care in which attitudes, behaviour, compassion, and dialogue are included [34]. Our findings also complement Anderberg et al.’s [12] suggestions about HPs’ general approach in palliative care. A general approach should involve individualized care, restore control, and provide respect, advocacy, and sensitive listening. Moreover, our results show that a general approach (e.g., “make decisions via mutual dialogues”, “avoid preconceptions”, and “use sensitive listening and empathy”) can be applied as specific care actions (e.g., “autonomy/control” and “care tenor”) to conserve dignity. This can be compared to Gallagher et al.’s [13] concept of “the culture of care” that includes the shared beliefs and values concerning the nature of care that prevail in the specific caring environment with a pursuit to let persons be involved in their own care. In line with our study, Gallagher et al. [13] state that “specific care activities” are about paying attention to a person’s individual preferences.

On the basis of the findings in the current study and the previous studies conducted in Scotland, Sweden, and international research publications [10, 15, 17, 27], we found no actual cultural differences in how to conserve dignity for persons with palliative care needs. Instead, the results in the current study expanded the multitude of care actions proposed for the different dignity-related concerns [19, 20]. This is positive, as conserving dignity is about respecting persons’ capacity and choices to live in a way that provides meaning to their lives and anchors their self-worth [8]. In vulnerable situations due to severe illness or frailty, the patient becomes dependent on others [6], such as HPs. A range of care actions increases the possibility to decide which ones are in line with the patient’s wishes. The literature particularly notes that the suggested care actions are to be reflected upon and decided in collaboration with the individual patient [16, 24]. This was also evident in the present study – all participants stressed the importance of all parties participating in care planning. They emphasised individualized care as well as mutual dialogue.

### Methodological considerations

In the deductive approach of analysis [30], the themes in Chochinov’s model of dignity [19, 20] were used as a framework. Data were reviewed for content suggesting care actions in relation to these themes. This implies our findings may be based on pre-understandings [35]. However, the search for care actions included inductive elements in which care actions emerged from the manifest content [36]. These care actions were then related to the model as a whole and the specific themes in the model. All authors were involved in the analysis to strengthen the trustworthiness of the results. All care actions found could be categorised under the themes. None fell outside the model and care actions were found for all dignity-related concerns. However, it was evident that some care actions were relevant to more than one theme and/or linked to each other. Such overlap could be interpreted as an incomplete analysis [35]. However, we interpret it as an endorsement that such care actions are part of a holistic and comprehensive approach to the complexity of dignity conserving care.

Effective nursing interventions require input from a variety of sources [28]. The suggestions of care actions in our study were therefore collected from patients with palliative care needs, SOs, nurses and physicians. Data were collected by a combination of individual interviews with patients and SOs, and focus groups with nurses and physicians. The integration of these two methods enhances data richness [32]. One could opine that the perspectives from SOs, physicians and, most importantly, patients are based on small sample sizes even if the total sample of 20 participants is quite large for a qualitative study [31]. This is important to consider when transferring the results [35]. However, the aim of our study was to suggest what dignity-conserving care actions consist of. Here our findings were in agreement with earlier studies with similar aims [10, 15, 17, 27]. Thus, it could be argued that the results are transferable to Swedish palliative care as well as to other countries with different palliative care settings. However, we suggest that it is still necessary to examine the adaptability to other cultures.

In future research, the DCI-SWE will be explored for its feasibility of use by municipality care nurses working in a palliative context. The intervention will then be evaluated by using multiple methods in line with guidelines for evaluation of complex interventions in nursing [37] and for the advancement of nursing practice [38].

## Conclusion

This study confirms that dignity conserving care is not just what is done for the patient, but also how the patient is viewed. This can be operationalised through specific and concrete care actions, as well as all-embracing attitudes and behaviours that conserve the patient’s dignity. In adapting the DCI to a Swedish context, we took into consideration the perspectives of older persons with palliative care needs, their SOs, nurses and physicians. Relevant care actions were added to the original ones in the Scottish DCI. The DCI-SWE can be used by nurses to care for patients with palliative care needs in general care. It has the potential to facilitate the nurses’ knowledge and to ensure evidence-based quality in palliative care. The effectiveness of the intervention and responses to it will be further researched.

## Abbreviations

DCI: Dignity Care Intervention
DCI-SWE: Dignity Care Intervention Swedish version
HPs: Health care professionals
PDI: Patient Dignity Inventory
SOs: Significant others
